# Clinical-epidemiological and laboratory profiles of severe *Schistosomiasis mansoni* infections at a university hospital

**DOI:** 10.6061/clinics/2017/e340

**Published:** 2018-09-14

**Authors:** Maria Cristina Carvalho do Espírito-Santo, Maíra Reina Magalhães, Naíma Mortari, Francisco Oscar de Siqueira França, Expedito José de Albuquerque Luna, Ronaldo Cesar Borges Gryschek

**Affiliations:** I Departamento de Molestias Infecciosas e Parasitarias e Laboratorio de Investigacao Medica 6, Faculdade de Medicina FMUSP, Universidade de Sao Paulo, Sao Paulo, SP, BR; II Instituto de Medicina Tropical, Universidade de Sao Paulo, SP, BR; III Centro Universitario UniFoa, Volta Redonda, Rio de Janeiro, RJ, BR

**Keywords:** *Schistosomiasis mansoni*, Clinical, Diagnosis, Treatment

## Abstract

**OBJECTIVE:**

Schistosomiasis remains a public health problem. This was a descriptive and retrospective study of 42 patients with a severe form of schistosomiasis who were admitted to the outpatient clinic of the Faculdade de Medicina da Universidade de São Paulo, São Paulo, in São Paulo, Brazil.

**METHODS:**

A data collection questionnaire was designed from the patient charts, and the following variables were evaluated: age, sex, place of birth, occupation, signs and symptoms of schistosomiasis, data from laboratory and imaging examinations, data regarding treatment outcomes, and the existence of comorbidities. Statistical analysis was carried out using Statistical Package for the Social Sciences 15.0 and Microsoft Excel 2003 software. The significance levels of the tests were fixed, accepting 5% type 1 error (α=0.05). Since this was a retrospective observational study, not all data were available for analysis.

**RESULTS:**

The mean age of the patients was 48.24 years; 57.1% were male. Statistically significant associations were observed between splenomegaly and thrombocytopenia (*p*=0.004) and between splenomegaly and leukopenia (*p*=0.046); however, only 4.5% of the patients had esophageal hemorrhage. Some patients received a specific treatment; of those, 42% took praziquantel, and 35.4% took oxamniquine. Nonspecific drug therapy was given as follows: 65% received propranolol, 90% omeprazole, and 43.6% aluminum hydroxide. The other treatments were as follows: 92.9% of patients underwent endoscopic treatment, 85% received sclerotherapy, and 62.5% used elastic bandages.

**CONCLUSION:**

This preliminary study presents a multidisciplinary outpatient follow-up associated with endoscopic and drug treatments that may be effective at preventing bleeding.

## INTRODUCTION

Schistosomiasis is endemic in 75 countries, constituting a great public health problem, and it is estimated that 230 million people are infected worldwide. Approximately 700 million people live in areas where they are at risk of infection with one or more of the following six species that may infect humans: *Schistosoma haematobium*, *Schistosoma intercalatum*, *Schistosoma japonicum*, *Schistosoma mansoni*, *Schistosoma mekongi* and *Schistosoma malayensis* 1.

Nevertheless, only *S. mansoni* has established itself in Brazil, and snails of the genus *Biomphalaria* spp are its intermediate host 2. It is estimated that approximately 6 million individuals are infected in Brazil, and approximately 25 million are exposed and are at risk of contracting the disease, with different prevalence rates in each state 3.

Data from the Ministry of Health (2008) showed that schistosomiasis causes more deaths (usually more than 500 per year) than dengue, visceral leishmaniasis (LD), and malaria 4.

The global impact of this disease is shown through data, such as the fact that 4.5 million disability-adjusted life years are lost per year, and there are 7000 deaths per year 5. It is important to highlight that there are debilitating sequelae, in addition to morbidity and mortality, that are indirectly associated with schistosomiasis, such as hepatic disease, portal hypertension, myelopathy, renal dysfunction with nephrotic syndrome, and pulmonary hypertension 6.

From the clinical perspective, schistosomiasis may be classified into an acute phase and a chronic phase. The acute phase starts with the penetration of cercariae into the skin and includes the passage of schistosomula through the lungs, the movement of adult worms into the veins of the portal system, and the onset of oviposition. The chronic phase represents the pathological and clinical manifestations from the location of the eggs in the tissues, the inflammatory reaction around them, and the action of antigens, whether from the adult worms or eggs, on tissues 7.

Chronic forms of the disease may trigger pulmonary, neurological, genital, and renal lesions in addition to portal hypertension, greatly affecting the patients' productive life. In addition, this disease brings a considerable risk of death, mainly due to esophageal hemorrhage. Thus, it is important to have specialized outpatient follow-up for patients with these clinical forms of *Schistosomiasis mansoni*.

The schistosomiasis outpatient clinic of the Hospital das Clínicas da Faculdade de Medicina da Universidade de São Paulo (HCFMUSP) started treating patients in the 1970s and currently follows approximately 50 hepatosplenic patients, both males and females. During the history of this outpatient clinic, there has been no systematic evaluation of the profile of these patients.

This study was necessary to analyze the epidemiological, clinical, and laboratory profiles of those affected by this disease to establish guidelines for care. The guidelines may have a positive impact on the quality of life of the patients.

## MATERIALS AND METHODS

### Study design

This is a descriptive and retrospective study that evaluated the charts of patients undergoing follow-up at the schistosomiasis outpatient clinic of the HCFMUSP, from January 2014 to December 2014.

The data used in the present study were transcribed from the patients' medical charts and entered into the study database.

### Inclusion criteria

• Being in the HCFMUSP schistosomiasis outpatient clinic during the study period January 2014 to December 2014.

• Presenting the chronic form of infection with *Schistosomiasis mansoni* and attending scheduled appointments every 6 months.

• Having laboratory tests performed in the HCFMUSP laboratory.

• Having EDA at least once a year performed at the Endoscopy Service HCFMUSP.

### Methods of diagnosis

A data collection questionnaire was designed from the patient charts, where the following variables were evaluated: age, sex, place of birth, occupation, signs and symptoms of schistosomiasis, data from laboratory and imaging examinations, data on treatment outcomes, and the existence of comorbidities.

In this study, we considered the hepatosplenic and pulmonary forms to be severe. In the hepatosplenic form, the signs observed were hepatosplenomegaly (periportal fibrosis) and portal hypertension with collateral portosystemic circulation, with or without ascites and with or without esophageal or gastric varices; hematologic signs of hypersplenism; and ultrasound images of "pipe-stem fibrosis" surrounding the portal veins. We also evaluated patients with signs of portosystemic hypertension and an absence of growths, either by splenectomy and/or decreased liver size due to advanced periportal fibrosis. In the pulmonary form (embolization and occlusion of the pulmonary capillary network by *S. mansoni* eggs), we observed signs of pulmonary hypertension, including breathlessness, fatigue, syncope, chest pain, and signs of right ventricle and tricuspid incompetence and peripheral edema.

The laboratory techniques used for this study were the Kato-Katz and Hoffman techniques, which confirmed positive results for *S. mansoni* eggs in the stool samples.

Since this was a retrospective observational study, not all data were available for analysis.

### Statistical analysis

The statistical analysis was carried out using Statistical Package for the Social Sciences (SPSS) 15.0 and Microsoft Excel 2003 software. The significance level of the tests was fixed, accepting 5% type 1 error (α=0.05).

The samples' characteristics were expressed as absolute and relative frequencies and as the means and standard deviations, when appropriate.

The associations between splenomegaly and platelet count and between splenomegaly and leukocyte count were evaluated.

To identify associations, the chi-square test was used. Fisher's exact test and the likelihood ratio test 8 were used when the sample was not large enough to apply the chi-square test.

### Ethics approval and consent to participate

Following the resolutions of CNS no. 196/96 and no. 346/05, a term of responsibility and confidentiality was developed regarding the individual information of the participants in this protocol.

The present research project was submitted to the Research Ethics Committee of the Department of Infectious and Parasitic Diseases of the Medical School of the University of São Paulo and to the Research Ethics Committee of Hospital das Clínicas (CAPPesq) of the Medical School of the University of São Paulo; the study was approved on 11/03/2015.

## RESULTS

The charts of 42 patients with severe forms of schistosomiasis who were undergoing follow-up at the outpatient clinic of the HCFMUSP in São Paulo, São Paulo were analyzed. In the study, 57.1% of patients were male (n=24/42), and 68.3% (n=28/41) had studied only up to the level of primary school. All the patients presented with the hepatosplenic form of the disease, as shown in Table 1.

The main origins of the patients observed included 38.1% (n=16/42) from Bahia, 19% (n=8/42) from Pernambuco, 16.7% (n=7/42) from Minas Gerais, 14.3% (n=4/42) from Alagoas, 4.8% (n=2/42) from Paraíba, 2.4% (n=1/42) from Piaui and 14.3% (n=4/42) from São Paulo. The patients from São Paulo lived in areas that had been endemic for schistosomiasis in their infancy.

Diabetes mellitus was the most frequent comorbidity (21.6% [n=8/37]), followed by systemic arterial hypertension (18.9% [n=7/37]), neoplasms (13.5% [n=5/37]), hepatitis C (12.2% [n=5/41]), hepatitis B (5.9% [n=1/17]), Chagas disease (5.4% [n=2/37]), heart failure, Hansen's disease, and human immunodeficiency virus (2.7% [n=1/37] each).

The mean age of the patients was 48.24 years, with a standard deviation of 13.32 years and a median of 47 years.

The patients sought medical care due to the presence of hepatosplenomegaly (33.3% [n=12/36]), abdominal pain/increased abdominal volume (22.2% [n=8/36]), anemia/pancytopenia (13.9% [n=5/36]), positive stool parasitological tests (13.9% [n=5/36]), bleeding (11.1% [n=4/36]), cough (2.8% [n=1/36]) and positive hepatic biopsy (2.8% [n=1/36]).

In this study, 11.9% (n=5/42) of patients were subjected to hepatic biopsy, 2.4% (n=1/42) to colon biopsy, and 2.4% (n=1/42) to lung biopsy.

The direct stool parasitological tests were positive for *S. mansoni* in 44% (n=15/24) of patients; these tests were carried out at the laboratory of the HCFMUSP.

Out of the patients with a positive test (n=15/24), 100% (n=15/15) were positive according to the Kato-Katz method, and 80% (n=12/15) were positive according to the Hoffman method.

As shown in Table 2, 96.9% (n=31/32) of these patients had used an antiparasitic drug. Out of those, 54% (n=13/24) used praziquantel, 46% (n=11/24) used oxamniquine, 90% (n=36/40) used omeprazole, 65% (n=26/40) used propranolol, and 43.6% (n=17/39) used aluminum hydroxide.

One of the patients presented with bleeding at the time of examination (Table 3).

Table 4 shows that 92.9% (n=39/42) of the patients were subjected to endoscopic treatment, 85% (n=34/40) to sclerotherapy and 37.5% (n=15/40) to elastic ligature.

In the present study, 57.1% (n=25/42) of patients had only esophageal varices and erosive gastritis observed on high digestive endoscopy, with 42.9% (18/42) of patients not presenting varices in their last examination. No gastric fundus varices were observed.

All endoscopies were performed at the Endoscopy Service HCFMUSP, following the same protocol of evaluation.

Table 5 shows that 95.2% (n=20/21) of patients with splenomegaly had thrombocytopenia, and 61.9% (n=13/21) of them also had leukopenia.

## DISCUSSION

In the present study, we analyzed the charts of 42 patients who were undergoing follow-up at the schistosomiasis outpatient clinic of the HCFMUSP from January 2014 to December 2014.

Despite its limitations, this was the first study of severe cases of infection with *Schistosomiasis mansoni* at the schistosomiasis outpatient clinic of the Clinical Division of Infectious and Parasitic Diseases of the HCFMUSP.

Most of the patients were male, as in the studies conducted by Coura et al. 8 and Drummond et al. 9. The mean age of the patients was 48.24 years, which was similar to the mean age in the study by Coura et al. 8, in which hepatosplenic and pulmonary forms were more frequent in the 20- to 40-year age group. In the study conducted by Drummond et al. 9, the mean age was 51 years (18-79). A possible explanation for this observation was the massive treatment program for schistosomiasis conducted at the end of the 1970s in Brazil, targeting school-age children. This fact might explain the mean age of the patients. This group of patients was probably not included in these mass treatment initiatives.

It is well known that socioeconomic and environmental factors play a fundamental role in human schistosomiasis 10,11. In our study, the mean level of education was elementary school. Other studies showed that this disease is related to a low level of education or no education 12,13 and to poverty; it is part of the cycle of disease and poverty 14.

Most of the patients in this sample came from the states of Bahia and Pernambuco, which are known hyperendemic regions of Brazil.

Regarding their clinical characteristics, most patients presented with the hepatosplenic form of schistosomiasis. Only one patient presented with the pulmonary form. This distribution is consistent with the findings in the study on morbidity conducted by Coura et al. 8, in which the predominant severe form was the hepatosplenic form 15.

Statistically significant associations were observed between the presence of splenomegaly and thrombocytopenia (*p*=0.004) and between splenomegaly and leukopenia (*p*=0.046). Patients with hepatosplenic schistosomiasis may have thrombocytopenia secondary to splenic sequestration 16-18.

In studies conducted in Africa, Boisier et al. 19 evaluated the complications of hepatosplenic schistosomiasis in different geographic areas. The presence of splenomegaly was observed in participants from the communities of Sahambano, Zazafotsy, and Voatavo. Melena, ascites, pallor, and ankle swelling were additional signs and symptoms observed in patients with the hepatosplenic form 20.

In the present study, 57.1% (n=25/42) of patients had only esophageal varices and erosive gastritis identified by upper endoscopy, with 42.9% (18/42) of patients not presenting with varices on the last examination. No gastric fundus varices were observed. Out of these patients, it was verified that 4.5% (n=1/22) had bleeding at presentation (in the last year of outpatient follow-up). All endoscopies were performed at the Endoscopy Service of the HCFMUSP, following the same protocol of evaluation. The study conducted by Rebouças 21 showed that gastrointestinal hemorrhage constitutes the most severe clinical manifestation and is partially proportional to the degree of portal hypertension 22.

Nonselective beta-blockers are commonly used to prevent bleeding caused by varices in patients with cirrhosis and esophageal varices. Recently, it was shown in a study conducted by Farias et al. 23 that the use of 40 mg of propranolol reduced the tension on esophageal variceal walls in over 20% of patients with schistosomiasis.

Only one of our patients who was using propranolol had bleeding while using this medication. Therefore, these data may suggest that propranolol was effective at preventing bleeding.

Sclerotherapy and endoscopic ligation are two endoscopic procedures used to treat esophageal varices, and both are effective in 80%–90% of cases, both in terms of the control of acute bleeding and in the prevention of recurrent bleeding 24.

In the present study, some of the patients underwent sclerotherapy, but elastic ligation was the most commonly applied technique. Other studies 24-28 reported that both sclerotherapy and elastic ligation are comparably effective in eliminating varices; however, elastic ligation is faster, has minor complications, and has lower recurrent bleeding rates, although it also has an increased rate of recurrent varices.

According to Widman et al. 29, splenectomy with azygoportal disconnection has rarely been indicated for the treatment of gastrointestinal hemorrhage resulting from esophageal varices in association with the portal hypertension due to the hepatosplenic form of infection with *Schistosomiasis mansoni*. It is the preferred surgery in most specialized health care centers in Brazil 30-32, and when it is associated with postoperative endoscopic sclerotherapy, it has good results for the prophylaxis of recurrent hemorrhage. In the present study, 13.2% of the patients underwent splenectomy, and this procedure was associated with esophagogastric devascularization.

Oxamniquine and praziquantel are drugs used in the treatment of schistosomiasis in Africa and in the Americas 33. In this study, our patients were treated with both drugs; some were administered praziquantel, while others were administered oxamniquine. We do not have information on a parasitological cure for these patients, since most of them already had a negative parasitological examination when they were admitted to our service, and just a few of them had stool exam results available in the health care medical data system. Stool examinations were negative in most of our patients because most patients had already been treated in other health care centers. These patients were referred to the outpatient clinic for follow-up of schistosomiasis portal hypertension.

This study had limitations because it was a retrospective study of medical records that was performed with a small sample within a limited time frame. The comparative changes related to the case series reflect the inconsistencies of these data in the medical records at the time they were recorded.

The complexity of the data presented demonstrates the complexity of the specialized care needed to maintain tertiary prevention and to reduce the morbidity and mortality of patients with severe schistosomiasis.

Despite the implementation of several control measures against infection with *Schistosomiasis mansoni* in Brazil over the past 40 years, there are still regions with high levels of endemicity and the presence of severe and debilitating forms of this helminthic disease. Affected individuals require specialized outpatient follow-up at a tertiary and/or quaternary level clinic in order to improve survival and their quality of life.

The Brazilian Association of Hepatology recommendations for the prevention and treatment of variceal bleeding in 2010 note the lack of data for the management of schistosomal portal hypertension, indicating that these recommendations are based on expert experiences. However, they suggest the efficacy of the use of nonspecific beta-blockers and endoscopic treatment as the main prophylaxis for esophageal variceal bleeding.

Despite its limitations, this preliminary study enabled the observation of the clinical profile of patients with the severe form of this helminthic disease.

Medical and endoscopic treatment in combination with clinical and laboratory follow-up have contributed to the prevention of bleeding.

Thus, we believe that the initial analysis carried out in this study may lead to further studies and that it shows the need for the implementation of an outpatient follow-up protocol for severe forms of *Schistosomiasis mansoni* infection to standardize its management and promote the equity of health care services.

We believe that integrated health actions may improve the quality of life of patients and promote social inclusion.

We believe that this study may contribute to filling data gaps, thereby improving the management of schistosomal portal hypertension, as noted by the guidelines of the Brazilian Association of Hepatology, 2010 34.

## AUTHOR CONTRIBUTIONS

Magalhães MR, Grysckek RC and Espírito‐Santo MC wrote the first draft of the manuscript. Magalhães MR, Gryschek RC, Mortari N, Luna EJ, Magalhães MR and Espírito‐Santo MC analyzed the data. Magalhães MR, Grysckek RC and Espírito‐Santo MC contributed to the writing of the manuscript. Magalhães MR, Grysckek RC, Mortari N, Luna EJ and Espírito‐Santo MC agreed with manuscript results and conclusions. Magalhães MR, Grysckek RC, Mortari N, Luna EJ and Espírito‐Santo MC jointly developed the structure and arguments of the paper. Magalhães MR, Grysckek RC, Mortari N, Luna EJ, Espírito‐Santo MC and França FO made critical revisions and approved final version. All authors reviewed and approved of the final manuscript.

## Figures and Tables

**Table 1:** Distribution of patient sex, education, and clinical form of schistosomiasis of those patients followed at the schistosomiasis clinic.

Variable	n	Valid %	CI (95%)	
			Inferior	Superior
Sex (n=42)*				
Male	24	57.1	42.2	72.1
Female	18	42.9	23.1	57.8
Education (n=41)*				
Elementary school	28	68.3	54	82.5
High school	13	31.7	14.5	46
**Hepatosplenic form**				
Yes	42	100	-	-
**Pulmonary form**				
Yes	2	5	0	11.8

* We have some information that is not available.

**Table 2:** Distribution of the use of antiparasitic drugs and other medication by patients followed at the schistosomiasis clinic.

Variable	n	Valid %	CI (95%)	
			Inferior	Superior
Antiparasitic drug (n=32)*				
Yes	31	96.9	90.8	100
Drug (n=24)*				
Praziquantel	13	54.2	34.2	74.1
Oxamniquine	11	45.8	18.7	65.8
Aluminum hydroxide (n=39)*				
Yes	17	43.6	28	59.2
No	22	56.4	-	-
Propranolol (n=40)*				
Yes	26	65	50.2	79.8
No	14	35	-	-
Omeprazole (n=40)*				
Yes	36	90	80.7	99.3
No	4	10	-	-

* We have some information that is not available.

**Table 3:** Prevalence of bleeding in patients followed at the schistosomiasis outpatient clinic of the Hospital das Clínicas, from January 2014 to December 2014, in São Paulo/SP.

Variable	Var Esof								Total		*p*
	Yes		CI (95%)		No		CI (95%)				
	n	%	Inferior	Superior	n	%	Inferior	Superior	n	%	
History of bleeding (n=40)*											0.799#
During treatment	2	9.1	0	21.1	1	5.6	0	16.2	3	7.5	
Without treatment	1	4.5	0	13.2	2	11.1	0	25.6	3	7.5	
No information regarding treatment	6	27.3	8.7	45.9	6	33.3	11.5	55.1	12	30	
No history of bleeding	13	59.1	38.6	79.6	9	50	26.9	73.1	22	55	
Total	22	100	-	-	18	100	-	-	40	100	
Current bleeding (n=40)*											>0.999**
Yes	1	4.5	0	13.2	0	0	0	0	1	2.5	
No	21	95.5	86.8	100	18	100	-	-	40	100	
Total	22	100	-	-	18	100	-	-	40	100	

Chi-square test; **Fisher's exact test; #likelihood ratio test.

* We have some information that is not available.

**Table 4:** Frequency of endoscopic and surgical treatments** of patients followed at the schistosomiasis clinic.

Variable	n	Valid %	CI (95%)	
			Inferior	Superior
Esophagogastric devascularization with splenectomy DAPE (n=38)**				
Yes	5	13.2	2.4	23.9
No	33	86.8	-	-
Endoscopic treatment (n=42)*				
Yes	39	92.9	85.1	100
No	3	7.1	-	-
Sclerotherapy (n=40)*				
Yes	34	85	73.9	96.1
No	6	15	-	-
Elastic ligature (n=40)*				
No	25	62.5	47.5	77.5
Yes	15	37.5	-	-

* We have some information that is not available.

** Surgical treatments - DAPE.

**Table 5:** Prevalence of associations between splenomegaly and the other variables found in patients followed at the schistosomiasis outpatient clinic of Hospital das Clínicas, from January 2014 to December 2014, in São Paulo/SP.

Variable	Var Esof								Total		*p*
	Yes		CI (95%)		No		CI (95%)				
	n	%	Inferior	Superior	n	%	Inferior	Superior	n	%	
Increased splenic vein diameter in abdominal ultrasonography (n=10)*											>0.999**
Yes	3	30	1.6	58.4	2	20	0	44.8	5	25	
No	7	70	41.6	98.4	8	80	55.2	100	15	75	
Total	10	100	-	-	10	100	0	0	20	100	
Platelets (mL/mm^3^) (n=21)*											**0.004#**
Reduced	20	95.2	86.1	100	8	50	25.5	74.5	28	75.7	
Normal	1	4.8	0	13.9	7	43.8	19.5	68.1	8	21.6	
Increased	0	0	0	0	1	6.2	0	18	1	2.7	
Total	21	100	-	-	16	100	-	-	37	100	
HB (g/dL) (n=21)*											0.899
Reduced	7	33.3	13.1	53.5	6	35.3	12.6	58	13	34.2	
Normal	14	66.7	46.5	86.9	11	64.7	42	87.4	25	65.8	
Total	21	100	-	-	17	100	-	-	38	100	
Leukocytes (µL/mm^3^) (n=21)*											**0.046**
Reduced	13	61.9	41.1	82.7	5	29.4	7.7	51.1	18	47.4	
Normal	8	38.1	17.3	58.9	12	70.6	48.9	92.3	20	52.6	
Total	21	100	-	-	17	100	-	-	38	100	

Chi-square test; **Fisher's exact test; #likelihood ratio test.

* We have some information that is not available.
